# Sustainability Claims of Nanoenabled Pesticides Require a More Thorough Evaluation

**DOI:** 10.1021/acs.est.3c10207

**Published:** 2024-01-23

**Authors:** Tom A. P. Nederstigt, Bregje W. Brinkmann, Willie J. G. M. Peijnenburg, Martina G. Vijver

**Affiliations:** † Institute of Environmental Sciences, Leiden University, 2300 RA Leiden, The Netherlands; ‡ National Institute for Public Health and the Environment, 3721 MA Bilthoven, The Netherlands

**Keywords:** nanoenabled pesticides, crop protection, environmental impact

Mitigation of pesticide use and risks has been center stage in developments in agriculture and food policies over the past year. While the European Parliament’s recent decision to reject the reformed Sustainable Use of Pesticides Regulation^1^ will undoubtedly be perceived by many as a disillusioning outcome to this end, the fierce debate that has surrounded its targets does emphasize a consensus about the need for novel means of crop protection that simultaneously are effective and result in minimal environmental impacts. Nanoenabled pesticides, i.e., pesticidal products with nanoscale active substances and carrier systems, are increasingly proposed to fit this purpose, and their favorable functionalities relative to non-nanoscale analogues have been abundantly highlighted in recent literature. While we recognize that some of the reported functionalities of nanoenabled pesticides may indeed hold potential for more efficient means of crop protection, we argue that claims regarding reduced environmental risks are often based on premises that insufficiently address their specific exposure and hazard profiles. We hereto provide an overview of key parameters that we believe should be accounted for more thoroughly when evaluating environmental risks or benefits associated with the use of nanoenabled pesticides.

## Classes, Properties, and Functionalities of Nanoenabled Pesticides

Nanoenabled pesticides can broadly be categorized into products in which nanomaterials serve as the active substance and those in which nanomaterials serve as a carrier system through which a conventional active substance is delivered (see ref (2) for an overview). Nanoscale active substances primarily consist of metal(loid) particles, while nanoscale carriers may also comprise (bio)polymers, clays, and carbon-based structures. Reported beneficial functionalities of both categories of nanoenabled pesticides include delayed and stimulus-dependent release of the active substance after application (i.e., extending or targeting exposure), improved adsorption and absorption (e.g., onto or into vegetative parts of crops or targeted organisms), and enhanced solubility and dispersibility (i.e., improved handling). Refinement of primarily the first two of these functionalities is often proposed to act as a double-edged sword. From a crop protection perspective, it could benefit input efficiency by maximizing the fraction of active substance that reaches the agricultural pest, while from an environmental perspective, it could mitigate undesired impacts on nontarget organisms by minimizing the amount of active substance that is displaced to adjacent ecosystems.

## Persisting Active Substances and Altered Exposure Profiles

**Figure 1:**
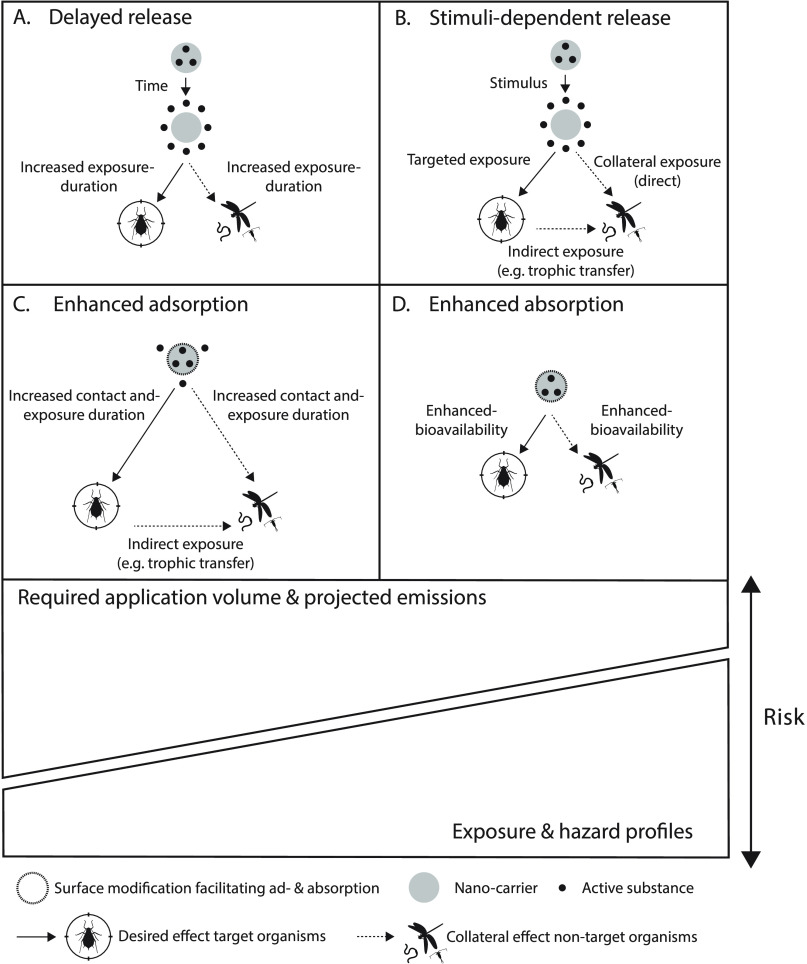
Although the reported functionalities of nanoenabled pesticides hold promise for reducing required application volumes, trade-offs with exposure and hazard profiles should be accounted for when claiming or evaluating benefits concerning environmental risks. Trade-offs are likely to emerge when specificity (i.e., toward target organisms) is not ensured, as summarized here.

Delayed-release mechanisms (i.e., facilitated by nanoscale carriers) aim to extend the availability of active substances to target organisms by reducing their rates of loss to processes such as hydrolysis, photolysis, and volatilization. As a consequence, their implementation could allow for a reduction in application volumes (and frequencies) of active substances and may concurrently decrease emissions to adjacent ecosystems. This decrease in emissions is often claimed to result in a reduction in associated environmental risks. However, these claims rarely acknowledge that in the absence of a mechanism that would retain the achieved persistence to the target site, this is likely to come at the cost of a similar increase in persistence of the (carrier-bound) active substance at nontarget sites (Figure 1A). A plethora of studies over the past years have demonstrated the ecological relevance of sublethal effects induced by chronic, low-dose exposure to pesticides.^3^ This underscores that even when net exposure concentrations of nontarget organisms would be decreased by utilizing delayed-release mechanisms, risk characterizations should equally account for resulting alterations in exposure times of nontarget organisms.

In the case of mechanisms of stimulus-dependent release, reductions in the fraction of active substance reaching nontarget organisms are primarily claimed to be achieved through functionalities that reduce runoff (e.g., by preventing release during precipitation, etc.) or maximize bioavailability in the presence of the target organism (e.g., in response to internally or externally excreted enzymes, etc.). It must be noted that such functionalities are unlikely to change the potential for nontarget organisms to be exposed through trophic transfer (Figure 1B), of which the relevance toward a variety of pesticides and nanomaterials has been well established.^4^ This argument holds for functionalities that aim to improve adsorption (i.e., onto crops or target organisms), as well (Figure 1C).

## Enhanced Bioavailability and Increased Information Requirements

In addition to the means by which the amount of active substance is maximized prior to reaching the target organism, the enhanced efficiency of nanoenabled pesticides may be accomplished via improved absorption after reaching the target organism (i.e., maximizing the fraction of the active substance reaching the internal molecular target). The mechanisms through which this may be achieved, such as tuning particle sizes and particle surfaces to facilitate transfer across biological barriers, are however rarely evaluated for their specificity toward target species. As such, there is currently little mechanistic ground on which to assume that commonly proposed mechanisms that enhance the bioavailability of nanoenabled pesticides toward target organisms do not equally do so toward (unintendingly) exposed nontarget organisms (Figure 1D).

The use of carrier systems and other co-formulants is no novelty to the pesticide industry, and there has been a long-standing debate regarding the extent to which these should be accounted for under environmental risk assessment frameworks applied for market approval. We argue that evaluations to this end for any nanoenabled pesticide (i.e., including those based on already approved active substances) should account for (i) the nanospecific properties of its constituents (regardless of being an active substance or co-formulant) and (ii) the potential alterations in nontarget exposure and hazard profiles of the active substance that may arise from functionalities of its formulation, as summarized in Figure 1. In practice, this would require fate and toxicity assessments of the individual constituents as well as of the formulated product. Considering that regulatory assessments are generally biased toward direct exposure and effects, we believe that to acquire a comprehensive understanding of potential nontarget impacts, fundamental ecotoxicological studies should focus on assessments of indirect exposure and effects via trophic interactions.

## Outlook

Various excellent reviews have provided overviews of parameters of concern to the environmental risk assessment of nanoenabled pesticides, some of which have addressed points described here and date back almost 10 years (see, e.g., ref (5)). Given recent developments toward achieving sustainability targets, which may include the commercialization of nanoenabled pesticides, we iterate the importance of considering trade-offs between usage volumes and exposure and hazard profiles that could concomitantly arise from their enhanced efficiency. We therefore contend that risk assessment of nanoenabled pesticides requires quantitative and mechanistic consideration of the specificity of obtained functionalities between target and nontarget organisms, including exposure durations and bioavailability, as well as indirect routes of exposure.
